# Use of autogenous onlay bone graft for uncontained tibial bone defects in primary total knee arthroplasty

**DOI:** 10.1186/s12891-017-1826-4

**Published:** 2017-11-29

**Authors:** Jung-Ro Yoon, In-Wook Seo, Young-Soo Shin

**Affiliations:** Department of Orthopedic Surgery, Veterans Health Service Medical Center, 61 Jinhwangdoro-gil, Gangdong-Gu, Seoul, 134-791 Korea

**Keywords:** Bone defect, Bone graft, Knee reconstruction, Knee replacement

## Abstract

**Background:**

The use of autogenous bone graft is a well–known technique for reconstruction of tibial bone defects in primary total knee arthroplasty (TKA). In cases where the size of the bone graft is inappropriate, the stability of bone graft fixation and subsequent bone graft to host bone incorporation may be compromised. We describe a simple and reliable technique of reconstruction in a proximal tibia bone defect at the time of primary TKA by using autogenous onlay bone graft (AOBG).

**Methods:**

Records were reviewed of 19 patients (mean age, 72 years) who underwent primary TKA using AOBG without the additional allogenous bone or metal augments, between August 2013 and August 2014.

**Results:**

Mean Knee Society score (KSS) in the 22 knees was significantly higher postoperatively than preoperatively (92 ± 4 vs. 30 ± 7, *P* < 0.001). The mean range of motion (ROM) in the 22 knees, which was 106 ± 12° preoperatively, improved to 112 ± 10° at last follow-up, but this this difference was not significant (*P* = 0.32). No migration of implants and presence of radiolucent lines at the bone cement-prosthesis interface were observed. Furthermore, the serial radiographs of 19 patients had a mean time of 3.2 months (range, 2.7–4.4 months) for solid union with cross trabeculation between the proximal tibial bone and graft.

**Conclusions:**

This simple AOBG supplement technique may biologically promote graft to host bone healing by enhancing fixation stability without the additional fixatives and assist the surgeon in managing the varying nature of uncontained bone defects.

**Trial registration:**

Trial registration number: KCT0002328, May 15, 2017.

## Background

A severe tibial bone defect in primary total knee arthroplasty (TKA) is one of the biggest challenges to treat for the surgeon, which can lead to a poorly balanced tibial component. Moreover, an uncontained defect is associated with a resultant angular deformity that is usually posterior and medial in a more than 20° varus knee from primary TKA [1]. The management of bone defects on the tibial aspect can vary depending on the size and location of the loss of bone, and it is necessary to consider other patient specific factors such as age, functional requirements, and bone quality. Various surgical options are available, including a thicker tibial bone resection, filling in the defect with methylmethacrylate cement, alone or with screws, and the use of metal augments [2, 3]. However, in certain situations, such as the presence of an uncontained, moderate, single condyle defect involving an area of 50% with a depth > 5 mm, a desirable surgical outcome may be difficult to achieve and bone graft may be needed. Although allogenous bone has now gained wide acceptance as a source of bone graft for primary or revision TKA owing to enhanced surgical and fixation techniques and increased functional outcomes, it has been reported to result in complications, such as risk of disease transmission, nonunion, collapse or resorption of the graft [4]. In overcoming these disadvantages, we describe a simple and reliable technique to reconstruct bone defects of the proximal tibia at the time of primary TKA by using autogenous onlay bone graft (AOBG) to ensure higher graft healing rates and lower infection rate without the use of an additional allogenous bone or metal augments. It was hypothesized that this method would achieve reliable clinoradiographic outcomes in these patients.

## Methods

### Inclusion criteria and enrolled patients

**Fig. 1:**
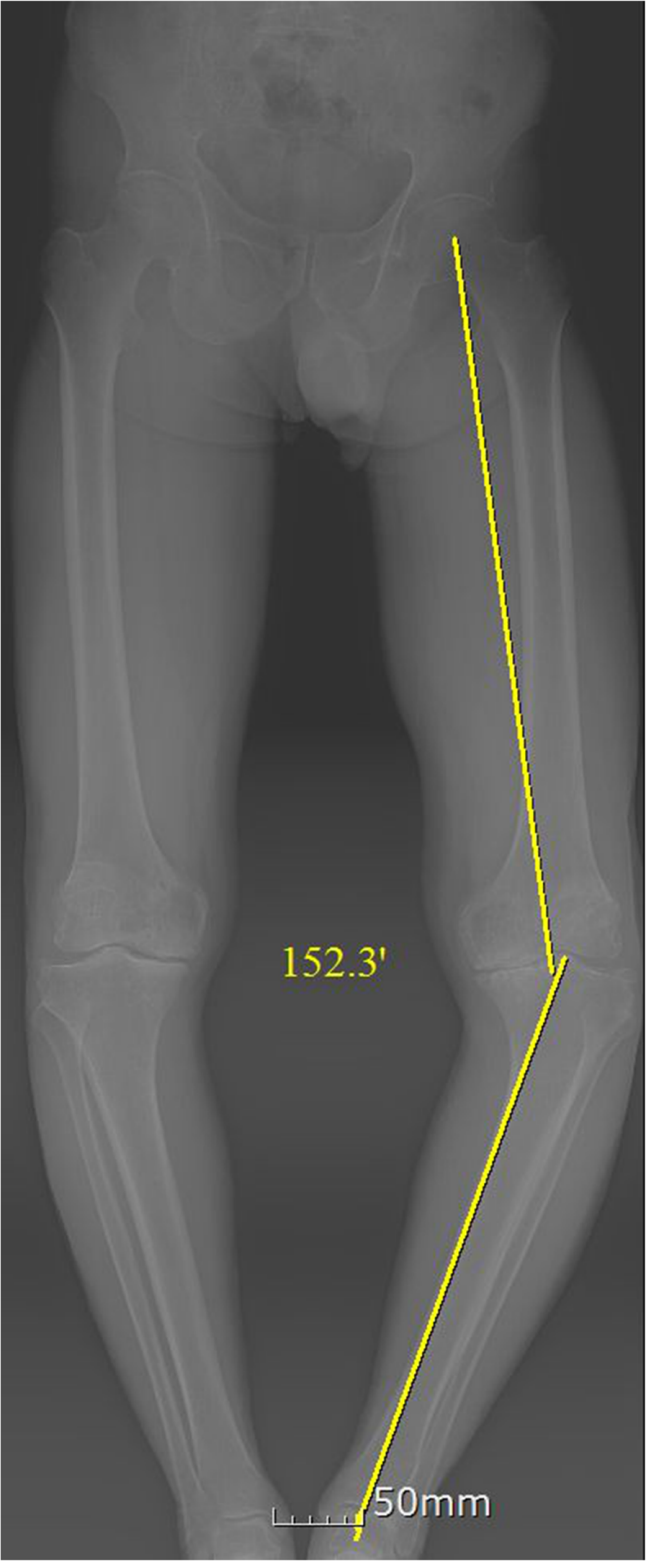
Preoperative long-standing anteroposterior radiograph of a patient with severe varus knee and marked medial bone loss

Between August 2013 and August 2014, 19 patients (22 knees) were performed primary TKA using AOBG. The present study included 19 patients (10 women and 9 men) with a hip-knee-ankle (HKA) of 20° or more on preoperative long-standing anteroposterior radiographs (Fig. 1). The diagnosis was degenerative osteoarthritis in all cases. Patients with valgus knees, rheumatoid knees and medial bone defects <5 mm were excluded. The average patient age was 72 years (range, 57–85 years) at the time of surgery. At follow-up evaluation, the patients were assessed clinically using Knee Society score (KSS) and range of motion (ROM). Postoperative radiographs and computed tomography (CT) scanning were analyzed for the presence of implant migration, defined as a vertical or angular displacement of the implant by 3 mm or 3°, respectively [5], and presence of radiolucent lines of ≥1 mm running parallel to the implant margins at the bone cement-prosthesis interface [6]. 2 orthopaedic surgeons measured the radiographic variables twice in all 19 patients, with a 2-week interval between measurements. This study was approved by the Ethics Committee of our institution (2016–11-001), and all patients provided written informed consent. The study was registered with the Republic of Korea Clinical Trials Registry (Identifier Number: KCT0002328).

### Surgical technique

The initial tibial proximal bone cut is performed using a standard tibial cutting guide. We reconstructed tibial defects with AOBG in primary TKA using a minimum of five steps.

**Fig. 2:**
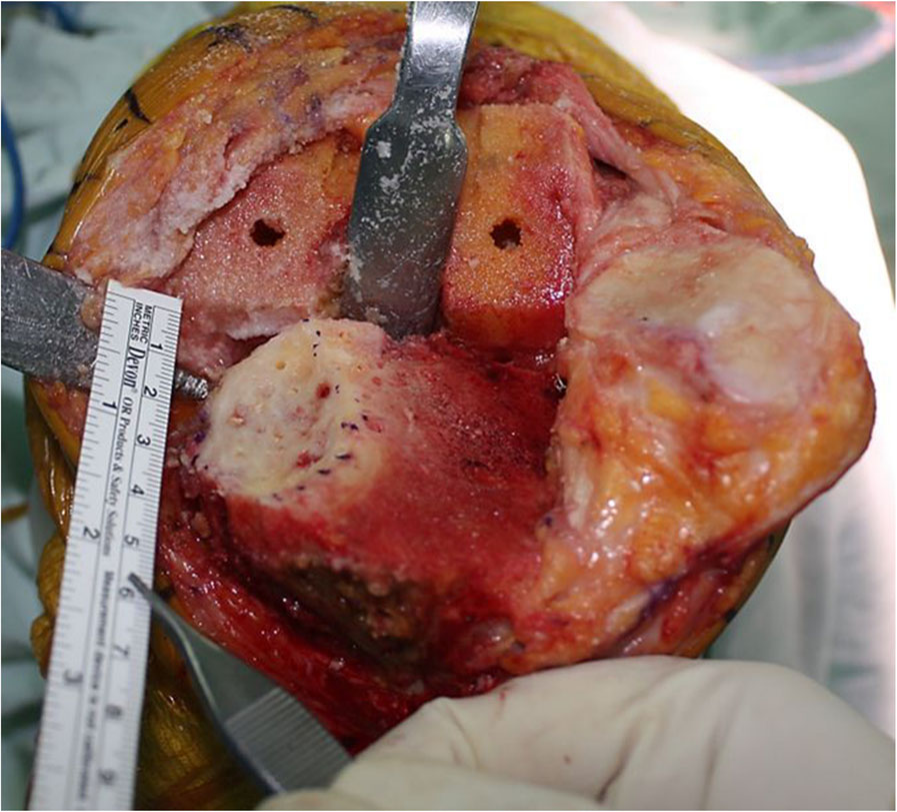
An intraoperative photograph showing delineation of severe tibial bone defects

**Fig. 3:**
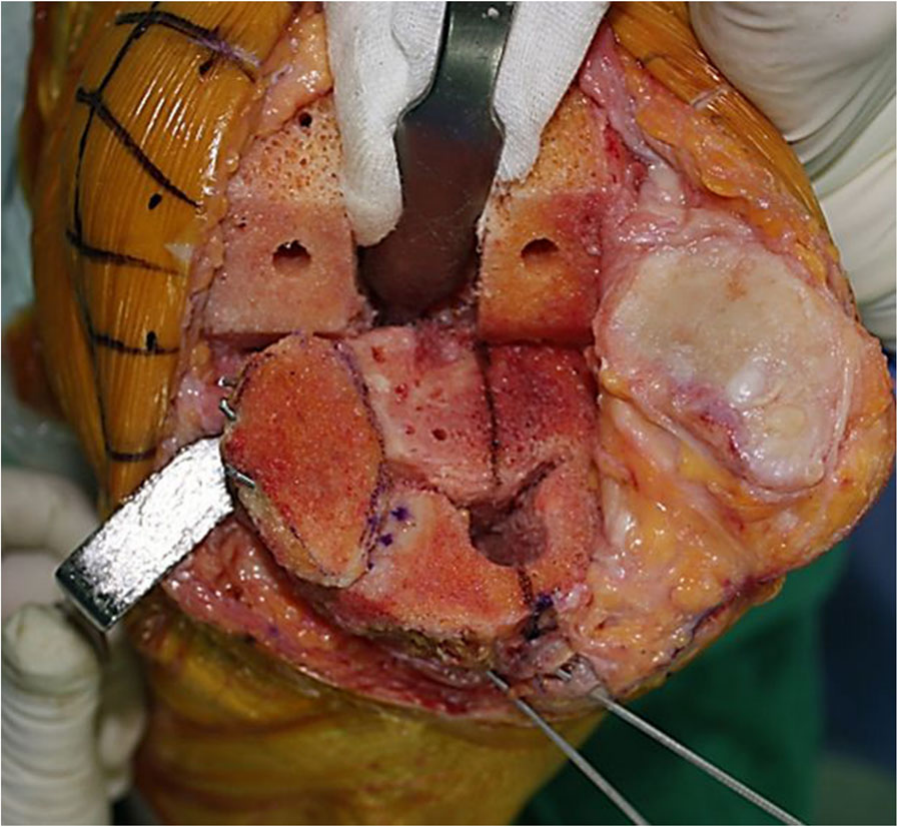
Uncontained defects of the posteromedial tibial condyle can be reconstructed with an autogenous onlay bone graft (AOBG) using provisional Kirschner wires

**Fig. 4:**
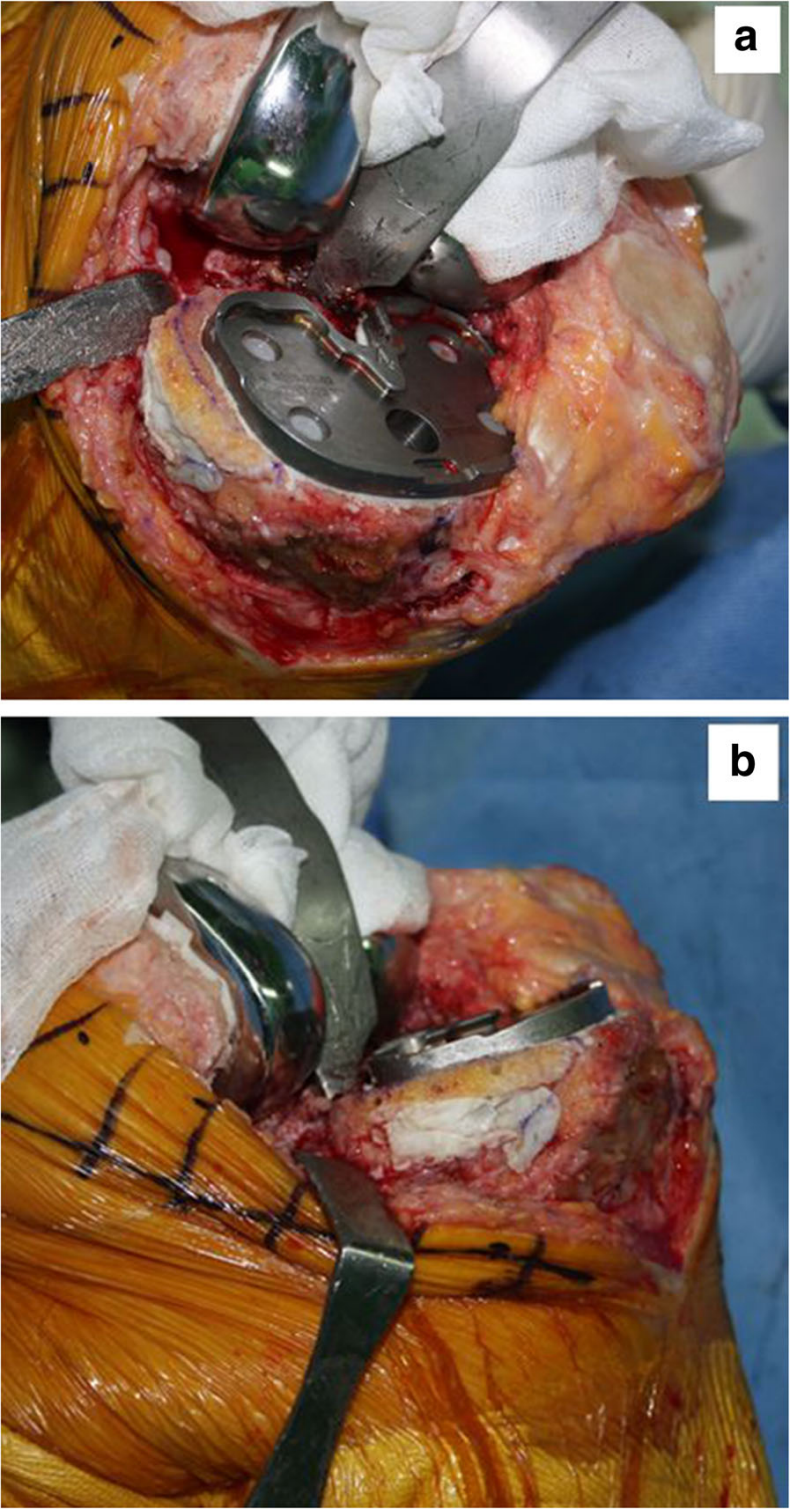
**a**, (**b**) An intraoperative photograph showing applying a small portion of cement to the medial surface of the tibia only along the line of the graft in order to preserve the soft tissue envelope at the fracture site

1. Measuring the defect size and recipient bed preparation
  The size of the tibial defect is measured using gauze shaped to fit the defect precisely after 8 to 10 mm of bone is resected from the lateral tibial condyle. Sizing is followed by predrilling and burring the recipient defect bed until bleeding occurs, otherwise unsuccessful incorporation and failure of the graft may occur (Fig. 2).
2. Bone graft preparation
  Autogenous bone from the posterior condylar bone (used for smaller defects) or proximal tibial bone (used for larger defects) is fashioned carefully depending on the size of the previously measured gauze using a combination of saw cuts, bony rongeurs, and a high speed burr so there is a match between the graft shape and the lesion to be filled, where the cartilage is peeled off and the subchondral bones are exposed. While creating an interference fit it is important to ensure that the bone graft diameter is not smaller than the diameter of the bone defect.
3. Bone graft fixation using provisional Kirschner wires
  Two 2 mm Kirschner wires are used to provisionally stabilize the AOBG to the host bone from back to front in parallel and aimed at a level below the anticipated joint line for the tibial component and in a position that does not interfere with the peg of the tibial component. In addition, the two Kirschner wires should pull forward as much as possible to make it easier to remove (Fig. 3).
4. Final fitting
  Once the AOBG is coapted, the portion of the AOBG that protrudes above the anticipated joint line is removed using an oscillating saw in a rough cut manner; a protrusion to the margin is trimmed by a bony rongeur. The AOBG frequently interrupts the approach to the medullary canal of the tibia. The medullary canal is first accessed with an oscillating saw and an osteotome, by which the surgeon creates a hole to advance through areas of the AOBG. This pre-made hole is expanded to pass intramedullary reamers into the canal.
5. Cementing and Kirschner wires removal
  It is of the utmost importance that cement not be allowed to permeate the gap between the graft and recipient defect bed. An excellent way to prevent this from occurring is to fill the empty space with any remaining graft fragments and apply a small portion of cement to the upper surface of the tibia along the line of the graft and the recipient defect bed (Fig. 4a, b). The Kirschner wires are withdrawn after fixation of the tibial component with cement that will harden.

Postoperative rehabilitation for our patient was the same as for patients without bone grafting in that weight-bearing and continuous passive motion is not limited. In cases with patients who undergo further release of medial structures such as the femoral origin of the MCL to obtain a rectangular mediolateral gap in some severe varus knees, a hinged brace is used for six weeks postoperatively [7].

### Statistical analysis

Statistical analyses were performed using SPSS statistical software version 20 (IBM Corp., Armonk, NY, USA). The preoperative and last follow-up KSS, ROM were analyzed using a paired-samples *t*-test. A *P*-value <0.05 was considered statistically significant. The reliabilities of measurements of radiographic alignment and time to union were determined by calculating the intraclass correlation coefficient (ICC) and the standard error of measurement, with ICC values >0.75, 0.4–0.75, and <0.4 representing good, fair, and poor reliability/accuracy, respectively. At an alpha level of 0.05 and a power of 0.8, we performed a post hoc power analysis to detect a mean difference of 5 points for KSS from before to after surgery. This study included 19 patients, with adequate power, to detect significant differences in KSS (0.823) from before to after surgery.

## Results

**Fig. 5:**
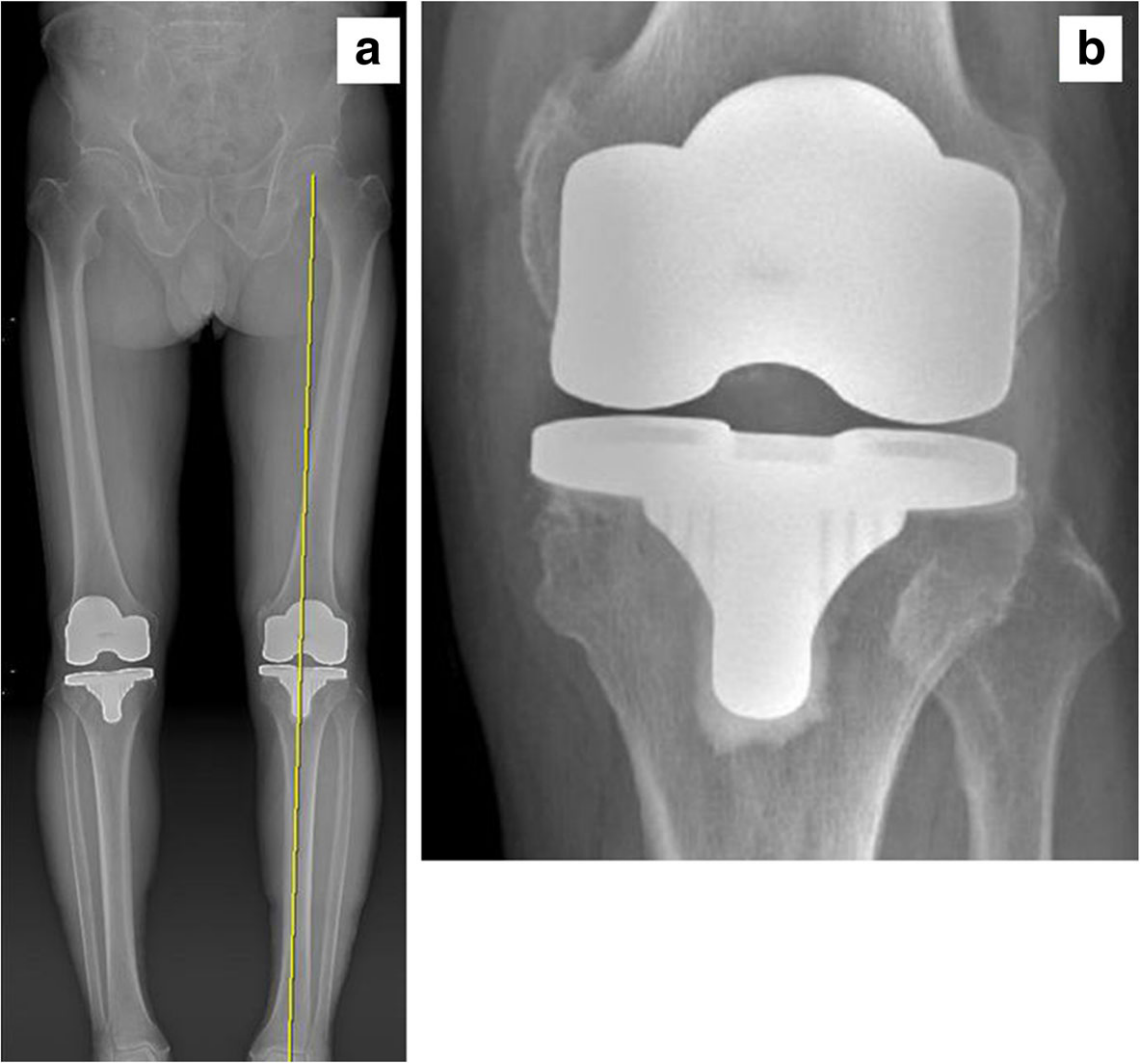
**a** Postoperative long-standing anteroposterior and (**b**) anteroposterior radiographs at year of an 72-year-old woman with the AOBG showed no migration of implants and presence of radiolucent lines at the bone cement-prosthesis interface

**Fig. 6:**
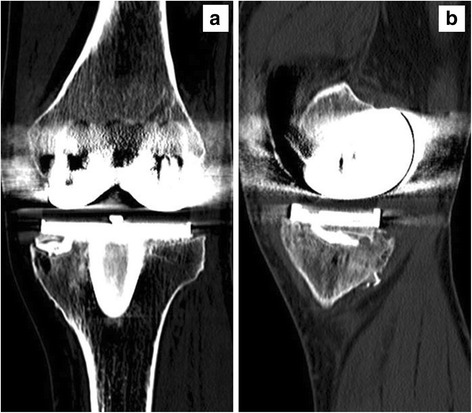
**a** Postoperative anteroposterior and (**b**) lateral CT scans at 1 year, demonstrating autograft incorporation with cross trabeculation between the proximal tibial bone and graft

The intra- and inter-observer reliabilities of the radiographic variables including time to union (0.769–0.0.856) and alignment (0.755–0.848) were satisfactory. The group was comprised of 10 women and nine men with a mean age of 72 years (range, 57–85 years) at the time of surgery. The mean depth of medial tibial defects, anteroposterior width, and mediolateral width were 12.0 mm (range, 9–20 mm), 33.2 mm (range, 28–44 mm), and 15.0 mm (range, 10–25 mm). The average follow-up period was 30.2 months (range, 24–36 months). Table 1 presents detailed data for uncontained defects in all cases. Mean KSS in the 22 knees was significantly higher postoperatively than preoperatively (92 ± 4 vs. 30 ± 7, *P* < 0.001). The mean ROM in the 22 knees, which was 106 ± 12° preoperatively, improved to 112 ± 10° at last follow-up, but this this difference was not significant (*P* = 0.32). None of patients experienced migration of implants and presence of radiolucent lines (Fig. 5a, b). Furthermore, the serial radiographs of 22 knees had a mean time of 3.2 months (range, 2.7–4.4 months) for solid union with cross trabeculation between the proximal tibial bone and graft (Fig. 6a, b). No a major complication, such as perioperative wound infection, was encountered in the present study.

**Table 1:** Baseline characteristics included in this study

Case No	Age (year)	Gender	Location	Source of grafting	Uncontained defect measurements (mm)			Follow-up (months)
					Depth	AP width	ML width	
1	77	F	PM	PCB	9	30	12	32
2	75	M	PM	PCB	10	32	15	28
3	82	F	PM	PCB	10	30	14	30
4	57	M	PM	PTB	14	35	16	24
5	65	M	PM	PCB	10	32	14	28
6	75	F	PM	PTB	15	38	16	32
7	72	M	PM	PCB	10	30	15	26
8	71	F	PM	PCB	10	30	12	30
9	68	M	PM	PTB	12	32	16	27
10	81	F	PM	PCB	10	30	15	28
11	74	F	PM	PTB	17	40	18	36
12	85	F	PM	PTB	14	36	15	34
13	72	M	PM	PTB	12	30	14	32
14	58	M	PM	PCB	10	32	12	30
15	74	M	PM	PTB	20	44	25	34
16	69	F	PM	PCB	10	30	14	28
17	79	F	PM	PTB	14	34	15	31
18	76	M	PM	PTB	18	40	20	29
19	77	F	PM	PCB	9	28	10	32
20	65	M	PM	PCB	10	32	14	30
21	59	F	PM	PTB	11	34	15	33
22	75	F	PM	PCB	10	32	12	30

*AP* anteroposterior, *ML* mediolateral, *PM* posteromedial, *F* female, *M* male, *PCB* posterior condylar bone, *PTB* proximal tibial bone

## Discussion

This simple technique to compensate for bone defects on the medial tibial condyle during primary TKA using the AOBG has proven to be quite effective regarding higher graft healing rates and lower infection rate.

The use of methylmethacrylate cement, independently or with screws, is a good option to fill contained defects ≤5 mm; additionally, bone grafts or metal augments may be used to offer support for tibial components for bone defects ≥5 mm [8]. Contained defects are ideal for impacted morselized bone grafts, which were found to be not recommended for repairing uncontained defects, because the cortical rim is considerable to ensure stability of the tibial component. In uncontained defects involving ≥50% of single tibial condyle, the use of structural allografts is recommended because they offer greater initial stability [9]. However, many shortcomings have been associated with the use of structural allografts, such as risk of disease transmission, nonunion, collapse or resorption of the graft [4, 10]. Another consideration is that they often require a longer period of limited weight bearing to allow for union of the grafts with the host bone. In addition, Whittaker et al. [11] described how to use metal augments in uncontained defects with moderate and severe bone loss ≥50% and ≥5 mm of the tibial condyle. However, there are some disadvantages, including further resection of bone to create a proper off-the-shelf augment fit and elevated cost. In an attempt to overcome these shortcomings, autogenous bone graft has been used for uncontained defects with marked bone loss of the tibial condyle; it is an advantageous method that improves graft union rates and bone stock preservation [12–14].

One study evaluating prerequisites for complete graft incorporation in 24 primary or revision knees reported that pertinent coverage of the graft by the component can prevent resorption of unstressed graft which may contribute to failure by collapse [1]. However, our study included one case of undersized tibial component on the cortical wall of the proximal tibia related to an our unintentional error during the initial learning curve. This suggests that limited coverage of the cortical wall of the proximal tibia may be related to early aseptic loosening especially with the standard tibial component. Nevertheless, we found no evidence of graft resorption or the need for revision surgery due to loosening of the tibial component. One reason for the better outcomes observed in the present study may be the use of autogenous bone obtained from posterior condylar bone or proximal tibial bone to augment bone defects on the medial tibial condyle, in cases where defect sizes are moderate with a depth of 5 to 20 mm in primary TKA, leading to a lower infection rate. However, the free-hand technique described earlier for the preparation of tibial bone defects may be somewhat technically demanding. Another factor that can explain the positive outcomes may be the soft tissue wall around the medial aspect of the proximal tibia, which can prevent the grafted bone from disintegrating after surgery. Furthermore, the AOBG can be harvested with little addition to the surgical time, and can be easily obtained without the additional fixatives, effectively creating the original shape of the medial tibial condyle in all cases by allowing the use of standard components for the primary system without tibial stem extenders. For additional fixatives, graft union rates have shown similar results across studies regardless of the presence of screws. One study classified tibial bone defects by their position and extent in 30 primary TKA cases without screw fixation treated with autogenous bone grafts. Nonunion between the graft and host bone occurred in one slant-peripheral-type case, resulting in 96.6% survivorship of autogenous bone grafts at 6.8 years [13]. In contrast, another study evaluating an autologous bone graft procedure attached the proximal portion of the tibial resection using two cannulated cancellous screws; the authors found that the screws were responsible for rigid initial fixation with a high rate of bony union at 6.6 years, which helped maintain long-term alignment [14]. Thus, we modified the surgical method to use temporary K wires instead of countersunk screws with more compression and less bone cement permeation under the graft. Additional surgical procedures, such as insertion of multiple screws may lead to fragmentation of the grafted bone, resulting in early failure of knee replacement [12].

We acknowledge the limitations of this article as it has a relatively small number of patients and short term follow-up study. However, it is not easy to find the appropriate indication of AOBG. In case of an uncontained, moderate, single condyle defect involving an area of 50% with a depth > 5 mm, surgeons may prefer use of allogenous bone or metal augments, because it does provide a simple way to reestablish the joint line without resecting the entire bone surface down to the level of the defect and offers the potential for early weight-bearing. Therefore, from a practical standpoint it is difficult to design and conduct randomized controlled trials comparing simple AOBG supplement technique and other techniques for a proximal tibia bone defect at the time of primary TKA.

## Conclusions

This simple AOBG supplement technique may biologically promote graft to host bone healing by enhancing fixation stability without the additional fixatives and assist the surgeon in managing the varying nature of uncontained bone defects, thereby preventing the risk of infection in primary TKA if this surgical technique is accurately performed.

## Abbreviations

AOBG: Autogenous onlay bone graft
CT: Computed tomography
KSS: Knee society score
ROM: Range of motion
TKA: Total knee arthroplasty

## References

[1] Kharbanda Y, Sharma M (2014). Autograft reconstructions for bone defects in primary total knee replacement in severe varus knees. Indian J Orthop.

[2] Dennis DA (1998). Repairing minor bone defects: augmentation & autograft. Orthopedics.

[3] Huten D (2013). Femorotibial bone loss during revision total knee arthroplasty. OrthopTraumatol Surg Res.

[4] Schmitz HC, Klauser W, Citak M, Al-Khateeb H, Gehrke T, Kendoff D (2013). Three-year follow up utilizing tantal cones in revision total knee arthroplasty. J Arthroplast.

[5] Chockalingam S, Scott G (2000). The outcome of cemented vs. cementless fixation of a femoral component in total knee replacement (TKR) with the identification of radiological signs for the prediction of failure. Knee.

[6] Shannon BD, Klassen JF, Rand JA, Berry DJ, Trousdale RT (2003). Revision total knee arthroplasty with cemented components and uncemented intramedullary stems. J Arthroplast.

[7] Lee SY, Yang JH, Lee YI (2016). A novel medial soft tissue release method for varus deformity during total knee arthroplasty: femoral origin release of the medial collateral ligament. Knee Surg Relat Res.

[8] Lonner JH, Lotke PA, Kim J, Nelson C (2002). Impaction grafting and wire mesh for uncontained defects in revision knee arthroplasty. Clin Orthop Relat Res.

[9] Dorr LD, Ranawat CS, Sculco TA, McKaskill B, Orisek BS (2006). THE CLASSIC: bone graft for Tibial defects in Total knee Arthroplasty. Clin Orthop Relat Res.

[10] Lee KJ, Bae KC, Cho CH (2016). Radiological stability after revision of infected total knee arthroplasty using modular metal augments. Knee Surg Relat Res.

[11] Whittaker JP, Dharmarajan R, Toms AD (2008). The management of bone loss in revision total knee replacement. J Bone Joint Surg Br.

[12] Ahmed I, Logan M, Alipour F (2008). Autogenous bone grafting of uncontained bony defects of tibia during total knee arthroplasty a 10-year follow up. J Arthroplast.

[13] Watanabe W, Sato K, Itoi E (2001). Autologous bone grafting without screw fixation for tibial defects in total knee arthroplasty. J Orthop Sci.

[14] Hosaka K, Saito S, Oyama T (2017). Union, knee alignment, and clinical outcomes of patients treated with autologous bone grafting for medial tibial defects in primary total knee arthroplasty. Orthopedics.

