# Correction to: Use of autogenous onlay bone graft for uncontained tibial bone defects in primary total knee arthroplasty

**DOI:** 10.1186/s12891-018-1939-4

**Published:** 2018-01-24

**Authors:** Jung-Ro Yoon, In-Wook Seo, Young-Soo Shin

**Affiliations:** Department of Orthopedic Surgery, Veterans Health Service Medical Center, 61 Jinhwangdoro-gil, Gangdong-Gu, Seoul, 134-791 Korea

## Correction

After the publication of this article [1] it came to the attention of the authors that there were 2 errors in the results section: 0.0.856 should be 0.856 and "this this difference" should have been "this difference".

## References

[1] Yoon (2017). Use of autogenous onlay bone graft for uncontained tibial bone defects in primary total knee arthroplasty. BMC Musculoskeletal Disorders.

